# A New Retest-Stable Tortuosity Metric for Retinal Vessel Analyses

**DOI:** 10.1167/iovs.65.12.30

**Published:** 2024-10-22

**Authors:** Samuel David Giesser, Ferhat Turgut, Amr Saad, Chiara Sommer, Yukun Zhou, Siegfried Karl Wagner, Pearse Andrew Keane, Matthias Becker, Delia Cabrera DeBuc, Gábor Márk Somfai

**Affiliations:** 1 Department of Ophthalmology, Stadtspital Zürich, Zurich, Switzerland; 2 Spross Research Institute, Zurich, Switzerland; 3 NIHR Biomedical Research Centre at Moorfields Eye Hospital NHS Foundation Trust, London, United Kingdom; 4 Institute of Ophthalmology, University College London, London, United Kingdom; 5 Department of Medical Physics and Biomedical Engineering, University College London, London; 6 Department of Ophthalmology, University of Heidelberg, Heidelberg, Germany; 7 Bascom Palmer Eye Institute, Miller School of Medicine, University of Miami, Miami, Florida, United States; 8 Department of Ophthalmology, Semmelweis University, Budapest, Hungary; 9 Gutblick Research, Pfäffikon, Switzerland; 10 iScreen 2 Prevent LLC, Miami, FL, United States

**Keywords:** tortuosity, retinal vasculature, fundus photography, mathematical modeling, deep learning

## Abstract

**Purpose:**

Existing retinal vessel tortuosity metrics lack standardization and retest reliability, hindering their clinical utility. Our study addresses this gap by introducing a novel metric, coined as the “vascular curvature index” (VCI), to enhance accuracy and consistency in biomarkers associated with medical conditions. We assess VCI's performance in terms of retest reliability in healthy subjects to transform early detection and monitoring approaches for various diseases.

**Methods:**

We recruited 44 patients for a single-session study, capturing fundus images before and after a five-minute break. Using AutoMorph, we generated vessel segmentation maps and evaluated retest reliability using multiple tortuosity metrics. The VCI was introduced and statistically compared to existing metrics. We performed a paired one-sided *t*-test to test for significantly improved retest reliability of our newly proposed metric. We analyzed distribution histograms, Fisher-Pearson coefficient of skewness, and correlation matrices for further insights.

**Results:**

VCI is the most retest-reliable metric, statistically surpassing other curvature metrics, except inverse spherical radius tortuosity. With a somewhat negatively distributed pattern (coefficient of skewness of −0.52), VCI exhibits the strongest correlation with the second most retest-reliable metric; inverse-radius-tortuosity (Pearson and Spearman correlation of 0.7 and 0.72, respectively). Its correlation with angle-tortuosity is lower (Pearson and Spearman correlation of 0.05 and 0.07, respectively).

**Conclusions:**

The VCI emerges as a highly retest-reliable metric with a relatively normal distribution in healthy patients. Further investigation is warranted to evaluate its clinical performance in real-world applications, potentially influencing proactive healthcare interventions and personalized treatment decision-making.

Metrics measuring blood vessel morphology, encompassing parameters such as vessel width, fractal dimension, and vessel tortuosity, play a pivotal role in understanding various medical conditions, and current vessel tortuosity metrics have significant associations with medical conditions such as chronic pulmonary diseases, diabetes, chronic venous diseases, and cardiovascular risk.^1^^–^^4^ These biomarkers have been extensively used to investigate changes in vessel morphology in different clinical settings, ranging from chronic obstructive pulmonary disease (COPD)^5^ and idiopathic pulmonary fibrosis^1^ to the classification of vessels during angiogenesis in oncology.^6^ Within oculomics, blood vessel tortuosity has already been proven to be associated with retinal vein occlusion, aging, diabetes, and hypertension.^1^^,^^7^^,^^13^^,^^14^ In spite of or perhaps because of these metrics’ popularity and wide application, no uniform definition of tortuosity as a biomarker exists.

In 2020, Krestanova et al.^8^ conducted a literature review to classify the proposed calculation methods for retinal vessel tortuosity applied to binary segmentation maps. The classified curvature metrics are designed to measure the two-dimensional curviness of a vessel as it moves across the retina and are based on the notion of curvature in Euclidean two-dimensional space, arc-over chord ratios with static or dynamically calculated stationary points, angle-based approaches that measure angular momentum across specific points and at different regions within the blood-vessel, rank-based approaches that attempt to classify a given image as more or less tortuous than another, and approaches examining the concavity between inflection points instead of curvature.^8^

Despite the acknowledged clinical relevance of tortuosity metrics, prior research has reported Pearson retest reliability for fundus-photography tortuosity ranging between 0.46 and 0.64 even within the same tortuosity calculation method.^9^ These limitations impede progress in clinical research and have the potential to lead to disparities in patient care. By enhancing the reliability of tortuosity evaluation, our proposed metric will contribute to improved diagnostic accuracy, treatment planning, and disease monitoring across a spectrum of medical specialties.

## Patients and Methods

Our recruited study group (*n* = 44) underwent a single imaging session where macula-centered fundus images were captured repeatedly in both eyes within a five-minute interval. Participants were drawn from a diverse pool comprising staff, patients, and visitors at Triemlispital. Individuals with recent ophthalmic surgery within the past six months or any history of ocular diseases were excluded from this group. Our recruited study group had a mean age of 41.3 years (range 20–70 years). We obtained informed consent from all participants and acquired 45° foveal-centered fundus images using a Zeiss Visucam Pro NM camera (Carl Zeiss Meditec AG, Jena, Germany) with the pupils in miosis.

We used the open-source tool AutoMorph to segment the vessel using the fundus images we obtained.^15^ AutoMorph processes fundus images, evaluating their quality upon input. We eliminated images of poor quality, whereas those of good quality undergo further analysis. AutoMorph generated a segmentation map outlining the vascular tree and optic nerve head for these high-quality images. Subsequently, each vessel within the image was classified as either an artery or a vein. We calculated the following tortuosity metrics for each segmentation map generated from our test-retest datasets.

### Arc-Over-Chord Ratio

To calculate the arc-over-chord ratio, we measured the arc length of blood vessels at discrete points along their paths. This calculation was done by summing the distances between consecutive points. The chord length, which represents the straight-line distance between the vessel's endpoints, was determined by Euclidean distance. The arc-over-chord ratio was computed by dividing the arc length by the chord length across fixed pixel distances.^8^ When writing a curve C as *C*(*t*) = (*x*(*t*), *y*(*t*)) with *t* ∈ [*a*, *b*] an interval in R, and x and y being smooth real functions, the used arc-over-chord ratio is a discretized version of the quotient of arc-length *s*(*C*) and chord-length *chord*(*C*).

$$\begin{array}{c} s\left(C\right)=\int_{a}^{b}\sqrt{x^{'}{\left(t\right)}^{2}+y^{'}{\left(t\right)}^{2}}dt,\;\;chord\left(C\right)=d_{2}\left(C\left(a\right),C\left(b\right)\right) \end{array}$$

where *d*_2_ represents the standard Euclidean distance function. With the discretized version of *s*(*C*) equating to

$$\begin{array}{c} s\left(C\right)=\sum_{t=a}^{b}\sqrt{x^{'}{\left(t\right)}^{2}+y^{'}{\left(t\right)}^{2}} \end{array}$$

### Distance Tortuosity

An arc-over chord ratio with a dynamic selection of points to construct the arc across the vessel.^7^ Points *a* and *b* represent the inflection points across the vessel's path.

### Angle-Based Tortuosity

We determined angle-based tortuosity by quantifying angular deviations along the trajectory of the blood vessels. We measured the angles at specific points along the vessel's path and calculated the mean of these angular deviations.^8^ For a curve *C*(*t*) with inflection points at $t\varepsilon \{t_{0},...,t_{n}\}$, the angular deviation is defined as

$$\begin{array}{c} \Delta_{\Theta }\left(i\right)=\pi -\arccos\left(a_{i}b_{i}/\left|a_{i}\right|\left|b_{i}\right|\right) \end{array}$$

with *a_i_* = *C*(*t_i_*) − *C*(*t_i_*/2 + *t*_*i* + 1_/2), *b_i_* = *C*(*t_i_*) + *C*(*t_i_*/2 + *t*_*i* + 1_/2) and iε{0,...,n-1}. Because retest reliability was improved by computing the weighted average with weights being the lengths of vessels between inflection points $\left(l_{0},...,l_{n-1}\right)$, the curvature metric of a vessel *s* is given by:

$$\begin{array}{c} \tau \left(s\right)=\sum_{i=0}^{n-1}l_{i}\Delta_{\Theta }\left(i\right)/n \end{array}$$

### Tortuosity Density

Tortuosity density, which assesses the degree of curvature within a localized region of the vessel, was calculated by evaluating the curvature of the vessel at specific intervals such that the intervals had constant sign curvature. Following the partition of the vessel *s* into regions $\left(C_{1},...,C_{n}\right)$, the formula is given by

$$\begin{array}{c} \tau \left(s\right)=\frac{n-1}{n}{\left(\sum_{i}chord\left(C_{i}\right)\right)}^{-1}\sum_{i=1}^{n}\left(\frac{s\left(C_{i}\right)}{chord\left(C_{i}\right)}-1\right) \end{array}$$

The procedure is described in detail in Grisan et al.^10^

### Squared Curvature Tortuosity

Squared curvature tortuosity was calculated by examining the arc-over-chord ratio curvature at discrete points along the vessel's path while also considering the height of the arc. The curvature at each point was computed, and a weighted average per vessel was calculated. This metric amplified the significance of curvature variations, emphasizing more pronounced geometric deviations. Defining the total curvature κ at point *t* as

$$\begin{array}{c} \kappa \left(t\right)=\frac{x^{'}\left(t\right)y^{''}\left(t\right)-x^{''}\left(t\right)y^{'}\left(t\right)}{{\left(y^{'}{\left(t\right)}^{2}+x^{'}{\left(t\right)}^{2}\right)}^{3/2}}, \end{array}$$

we define the total squared tortuosity of a vessel as

$$\begin{array}{c} \tau \left(s\right)=\int_{a}^{b}\left|\kappa \left(t\right)\right|dt \end{array}$$

A full description, including a description of linear interpolation and smoothing operations, may be found in Hart et al.^11^

### Inverse Spherical Radius Tortuosity

To compute inverse spherical radius tortuosity, the radius of curvature at each point along the vessel was determined. The inverse of these values was then calculated, and the mean of these inverses provided the inverse spherical radius tortuosity.^12^

### The Newly Proposed VCI Metric

**Figure 1.**
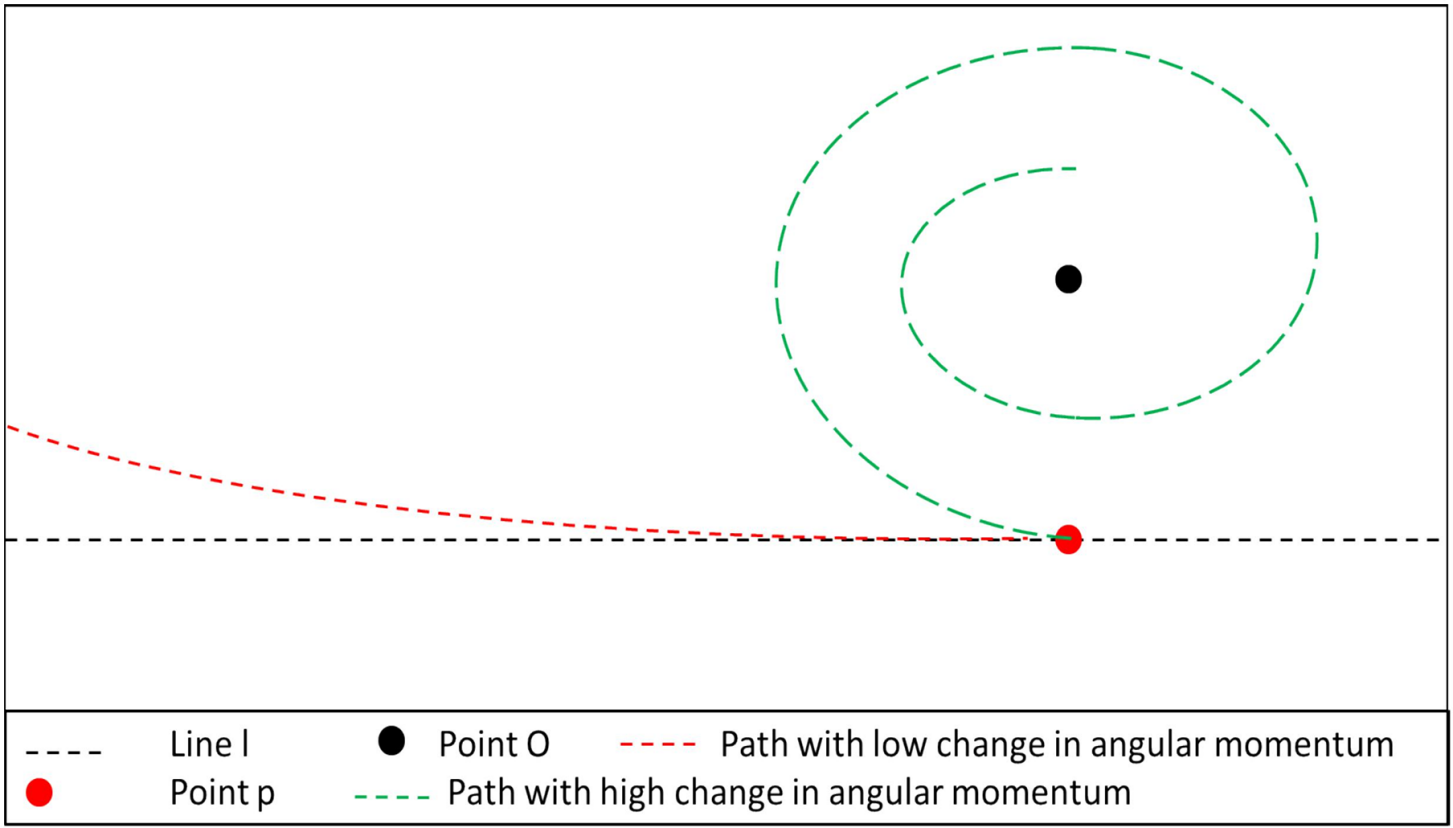
Examples of trajectories of point p assuming constant speed at high and low changes of angular momentum with respect to point O.

The VCI metric is a proprietary metric based on a measurement of change in angular momentum. Angular momentum is given by *L* = *I*ω with *I* the moment of inertia and ω the angular velocity. Assuming a simplified example of a point p rotating around an axis, we can write the inertia *I* as *I* = *r*^2^*m* where the radius *r* may be expressed as a function of time, and *m* being the mass of the examined point in space and the angular velocity, in this setting, equating to ω = *v*/*r*. Therefore, in this specific example, the change in angular momentum is given by $dL/dt=mv(r^{'}\left(t\right))$. Figure 1 illustrates the differences between a high and low change in angular momentum: Point *p* is traveling across the line *l*. With respect to point *O*, we apply a constant change in angular momentum, such that the rate of increase of the distance between *O* and *p* is greater than the change in angular momentum. Assuming constant speed, the result is a deviation from the natural path along line *l*, where the distance between the points *p* and *O* increases and is shown in the red path. As shown in the green path, a sufficiently high angular momentum change will result in point *p* circling in concentric circles around point *O*. VCI is designed to resist segmentation inaccuracies that may arise during the segmentation, thresholding, and skeletonization process.

**Figure 2.**
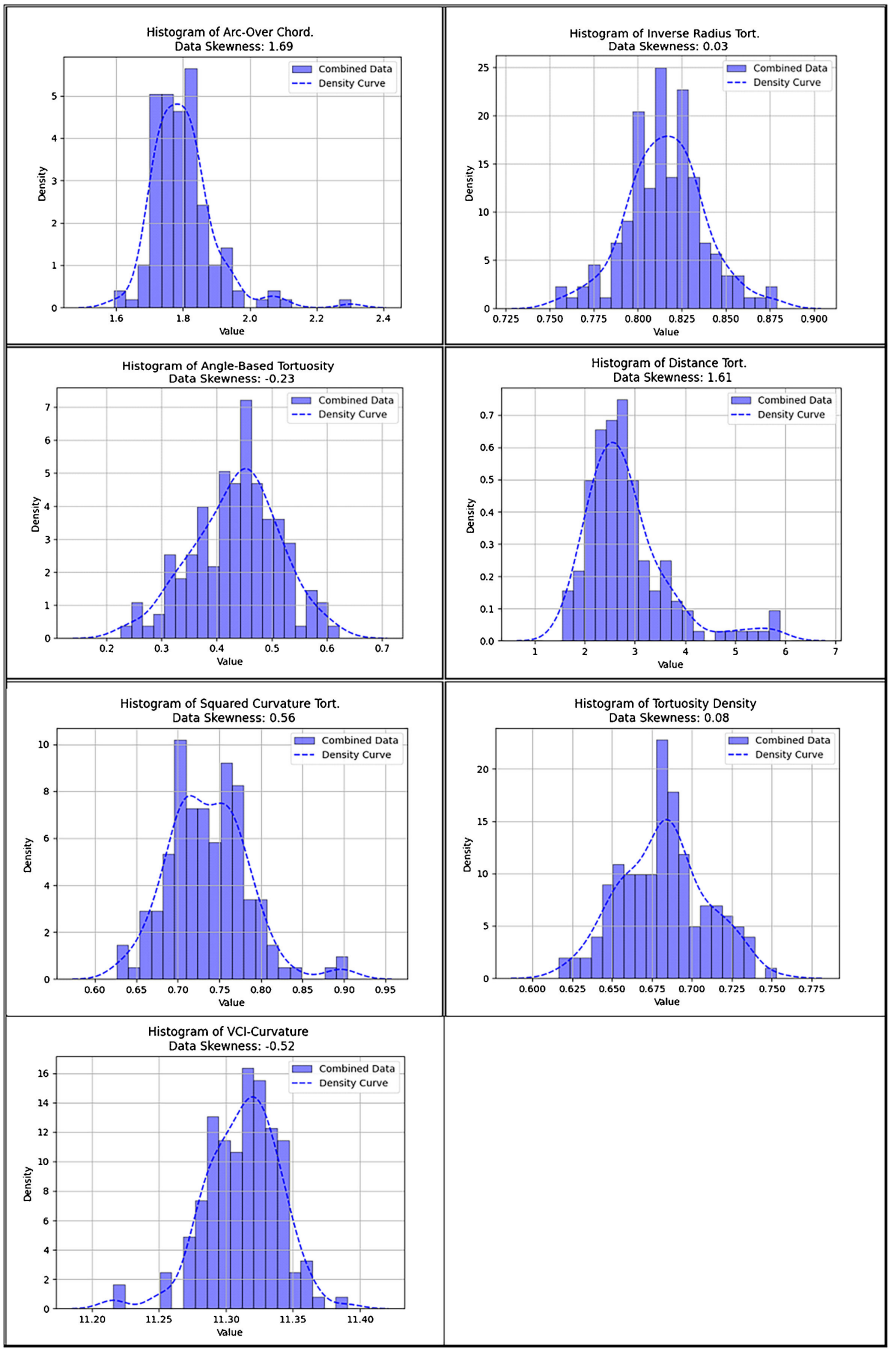
Histograms of the different curvature metrics: arc-over-chord ratio, inverse radius tortuosity, angle-based tortuosity, distance tortuosity, squared curvature tortuosity, tortuosity density, and VCI-curvature. Data skewness is given by the Fisher-Pearson coefficient of skewness.

We calculated the Spearman and Pearson correlation coefficients for each examined metric for arteries, veins, and vessels for our statistical analysis. Next, we performed a paired one-sided *t*-test to determine whether there was a statistically significant improvement in our VCI metric regarding retest stability versus the established vessel tortuosity biomarkers. We provided histograms of our examined tortuosity metrics and measured each metric's Fisher-Pearson coefficient of skewness (Fig. 2). Finally, we estimated the Pearson and Spearman correlation between our VCI and other tortuosity metrics to evaluate its cross-validity. As additional information, we provide sample visualization heatmaps of VCI tortuosity.

## Results

Tables 1 and 2 summarize the Pearson and Spearman coefficients between tests, respectively. Using a paired one-sided *t*-test, we found that the VCI metric has a significantly lower absolute difference between test and retest in Box-Cox-transformed z-scores to all other metrics except inverse radius tortuosity. The *p* values of the test-retest of the tortuosity metrics are shown in Table 3, which indicates that the VCI metric is significantly more stable on retest than every other examined metric in this article, except for inverse radius tortuosity.

**Table 1.** Spearman Coefficients Between Test and Retest

	Spearman Test-Retest Correlation		
	Arteries	Veins	Vessels
Arc-over chord	0.46	0.50	0.48
Angle-tortuosity	0.80	0.49	0.62
Tortuosity density	0.55	0.56	0.80
Squared curvature tortuosity	0.67	0.57	0.78
Distance tortuosity	0.73	0.66	0.73
Inverse-radius tortuosity	0.80	0.86	0.82
VCI metric	0.92	0.86	0.87

**Table 2.** Pearson Coefficients Between Test and Retest

	Pearson Test-Retest Correlation		
	Arteries	Veins	Vessels
Arc-over chord	0.67	0.53	0.60
Angle-tortuosity	0.59	0.68	0.62
Tortuosity density	0.60	0.61	0.61
Squared curvature tortuosity	0.45	0.71	0.66
Distance tortuosity	0.53	0.80	0.75
Inverse-radius tortuosity	0.87	0.85	0.86
VCI metric	0.86	0.94	0.89

**Table 3.** *P* Values of One-Sided Paired *t*-Test of the Box-Cox-Transformed *z*-Scores of the Absolute Difference Between Test and Retest of Metrics

*P* Value Versus	Arc-Over-Chord	Distance Tortuosity	Angle-Tortuosity	Tortuosity Density	Squared Curv. Tort.	Inverse Radius Tortuosity
VCI metric	<0.001	0.007	<0.001	<0.001	0.003	0.14

**Figure 3.**
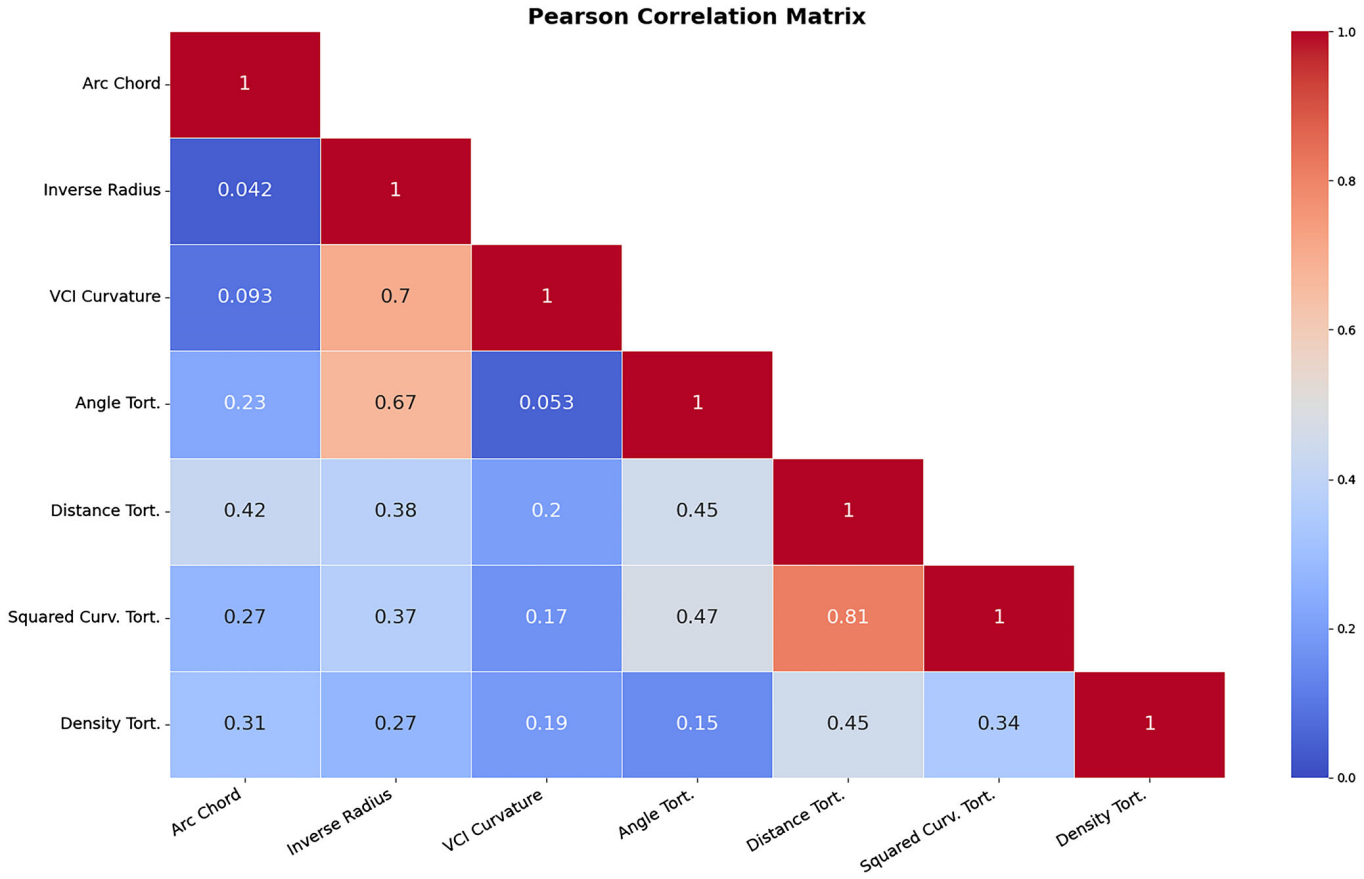
Pearson Correlation matrix between our examined tortuosity metrics.

**Figure 4.**
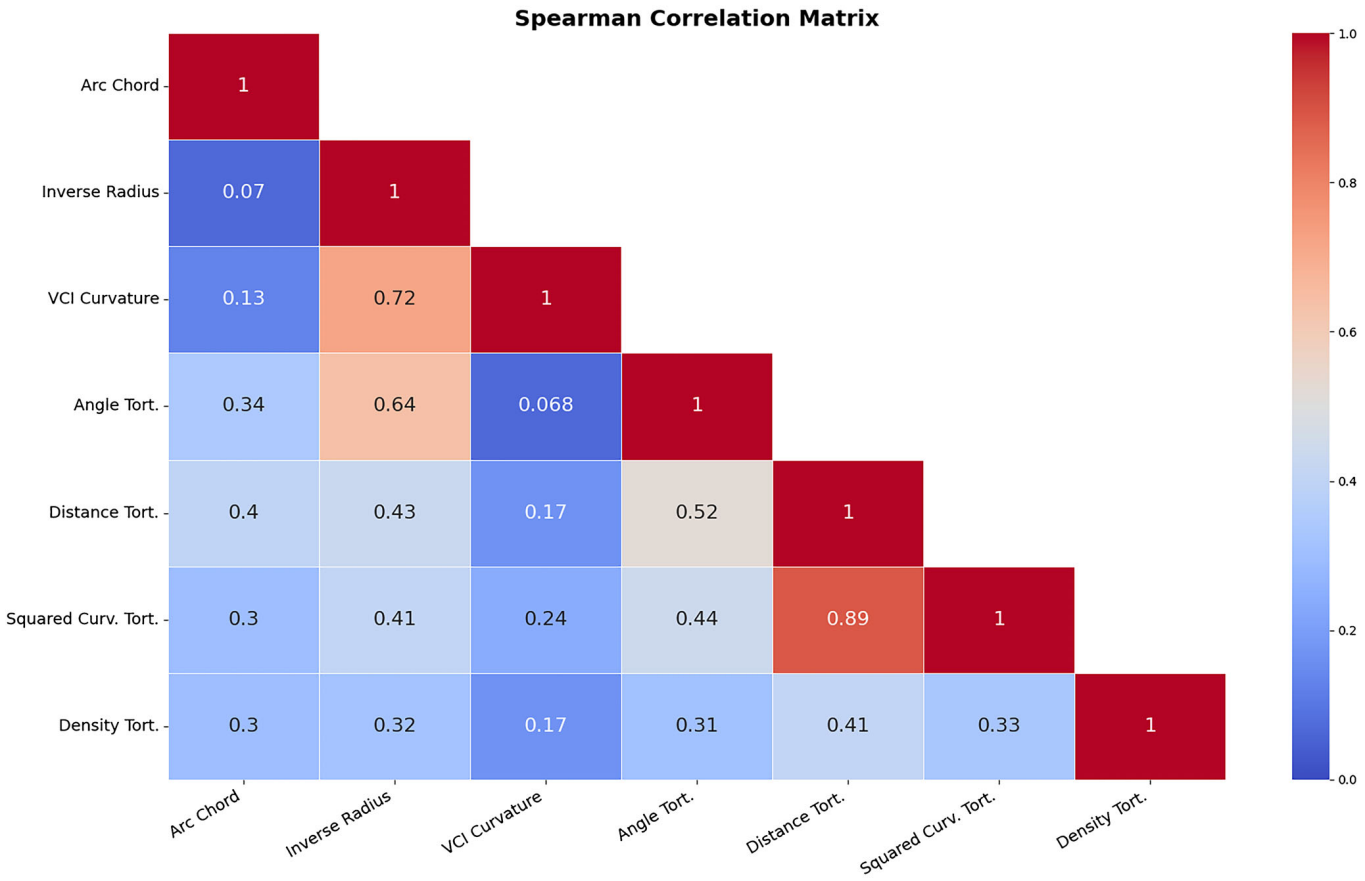
Spearman Correlation matrix between our examined tortuosity metrics.

**Figure 5.**
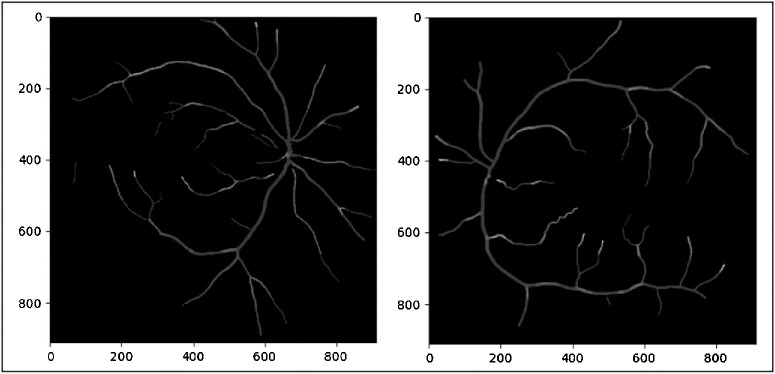
Sample heatmaps of the VCI metric. Light regions correspond to relatively high regions of tortuosity darker regions correspond to relatively low regions of tortuosity.

Figures 3 and 4 provide Pearson and Spearman correlation coefficients of the different tortuosity metrics examined. The highest Pearson correlation coefficient between tortuosity metrics was measured between distance and squared curvature tortuosity with a correlation coefficient of 0.81. The lowest recorded Pearson coefficient was between the arc-over-chord ratio and inverse radius tortuosity, with a Pearson correlation coefficient of 0.042. The highest and lowest correlation measures were the same when measuring correlation with the Spearman coefficient with Spearman coefficient values of 0.89 and 0.07, respectively. VCI curvature was most closely correlated with inverse-radius tortuosity; this was the second strongest correlation between two tortuosity metrics by Pearson and Spearman correlation. Figure 5 provides some sample results of heatmaps of the VCI-metric.

## Discussion

In this study, we have introduced a novel retest-stable tortuosity metric and assessed its performance in healthy subjects. Compared to standard tortuosity metrics, the VCI demonstrated significantly improved retest reliability. Specifically, in our sample of 44 subjects, the VCI metric had significantly higher retest stability than all examined metrics (*P* < 0.05) except for inverse radius tortuosity.

The VCI metric strongly correlated to the second most reliable metric, inverse radius tortuosity, with Pearson and Spearman correlation coefficients of 0.7 and 0.72, respectively. By enhancing retest reliability, our proposed VCI metric addresses a fundamental limitation in existing tortuosity quantification. Using our methodology, we significantly improved the reproducibility of AutoMorph metrics. This improvement will enable more accurate diagnostics, disease monitoring, and treatment evaluations across various medical disciplines relying on precise vessel morphology measurements.

Although a significant contribution, our findings should be considered with certain limitations. First, variability in segmentation quality may still affect tortuosity calculations. Second, metric specificity needs to match the clinical context. Although we assessed various metrics, further optimization is required for distinct medical applications. Third, we examined short-term reliability; long-term stability remains to be investigated. Consequently, the exploration of long-term retest reliability and its implications for disease progression and treatment monitoring were deemed beyond the immediate purview of our research. Additionally, it is important to acknowledge that our recruited patients underwent screening to ensure the absence of ocular disease. Consequently, we lack information regarding the replicability of similar results in fundus photography among patients with conditions such as diabetic retinopathy, vitreous hemorrhage, age-related macular degeneration, or the presence of vitreous haze. Finally, performance may differ across imaging modalities: VCI validation is necessary for modalities beyond fundus photography used here. For instance, metrics that prove informative in optical coherent tomography may not exhibit comparable utility in fluorescein angiography or other modalities that may be capable of tracking vessels in three-dimensional space whereas VCI measures curvature across a two-dimensional fundus. Therefore acknowledging the variability in clinical significance across different imaging techniques is imperative. Translating our findings to diverse imaging modalities would necessitate thorough pre-validation procedures. In demonstrating substantially improved retest stability over leading alternatives, our proposed VCI tortuosity metric represents a valuable new tool for precise vessel morphology quantification.

## Conclusions

Retest reliability assessment is fundamental in ensuring the consistency and robustness of tortuosity metrics for blood vessels. In this study, we analyzed a diverse array of tortuosity, such as arc-over chord ratio, angle-tortuosity, tortuosity density, squared curvature tortuosity, distance tortuosity, inverse-radius tortuosity, and our VCI metric, in arteries, veins, and the overall vascular network.

The results highlight the significant variations in retest reliability among the tortuosity metrics. Notably, the VCI exhibited the highest retest reliability across all categories, indicating its potential as a consistent and dependable metric for assessing vascular tortuosity. This metric's superior performance suggests its applicability in clinical settings, where precise and reproducible measurements are paramount. However, it is also a metric that showed a low overall correlation with established curvature metrics both in Spearman and Pearson correlation coefficients. By contrast, inverse radius tortuosity showed slightly, but not statistically significantly, poorer retest reliability but higher correlation with established curvature metrics. Nevertheless, there was a moderate to good correlation between inverse radius tortuosity and VCI metrics (Pearson and Spearman coefficients 0.7 and 0.72, respectively). Further research is warranted to explore the clinical relevance of the VCI metric and its potential contributions to diagnostics and disease monitoring in various medical specialties. The findings of this study contribute to the ongoing efforts to enhance the accuracy and reliability of tortuosity metrics, ultimately advancing clinical decision-making and patient care. Our novel methodology significantly improved the reproducibility of AutoMorph metrics, and it is an essential step toward screening and risk prediction of various retinal diseases.
